# Neuroinflammation Based Neurodegenerative In Vitro Model of SH-SY5Y Cells—Differential Effects on Oxidative Stress and Insulin Resistance Relevant to Alzheimer’s Pathology

**DOI:** 10.3390/ijms26146581

**Published:** 2025-07-09

**Authors:** Csenge Böröczky, Alexandra Paszternák, Rudolf Laufer, Katinka Tarnóczi, Noémi Sikur, Fruzsina Bagaméry, Éva Szökő, Kamilla Varga, Tamás Tábi

**Affiliations:** 1 Department of Pharmacodynamics, Semmelweis University, 4 Nagyvárad tér, H-1089 Budapest, Hungarypaszternak.alexandra@semmelweis.hu (A.P.); laufer.rudolf@semmelweis.hu (R.L.); tarnoczi.katinka.timea@semmelweis.hu (K.T.); bagamery.fruzsina@semmelweis.hu (F.B.); tabi.tamas@semmelweis.hu (T.T.); 2 Center for Pharmacology and Drug Research & Development, Semmelweis University, 26 Üllői út, H-1085 Budapest, Hungary

**Keywords:** neuroinflammation, insulin resistance, Alzheimer’s disease, microglia

## Abstract

Neuroinflammation is a key process in Alzheimer’s disease (AD). We aimed to examine the development and evaluation of a comprehensive in vitro model that captures the complex interplay between neurons and immune cell types. Retinoic acid-differentiated SH-SY5Y neuroblastoma cells exposed to LPS-conditioned media (CM) from RAW264.7 macrophages, BV2 microglia, and HL60 promyelocytic cells differentiated into neutrophil- or monocyte-like phenotypes were analyzed. The effects of CM containing inflammatory factors on neuronal viability and function were systematically evaluated. Neuronal oxidative stress, mitochondrial function, autophagy and protein aggregates were analyzed. The involvement of insulin resistance was studied by assaying glucose uptake and determining its IC_50_ values for cell viability improvement and GSK3β phosphorylation. After short-term exposure (3 h), most inflammatory CMs induced peroxide production in neurons, with the strongest effect observed in media from DMSO- or RA-differentiated HL60 cells. Mitochondrial membrane potential was markedly reduced by LPS-stimulated BV2 and HL60-derived CMs. Prolonged exposure (72 h) revealed partial normalization of oxidative stress and mitochondrial membrane potential. Glucose uptake was significantly impaired in cells treated with LPS-activated RAW264.7, BV2, and DMSO-differentiated HL60 cell media, while insulin partially rescued this effect, except for the CM of BV2 cells. Notably, insulin IC_50_ increased dramatically under LPS-treated BV2 cells induced inflammation (35 vs. 198 pM), confirming the development of insulin resistance. Immune cell-specific inflammation causes distinct effects on neuronal oxidative stress, mitochondrial function, protein aggregation, insulin signaling and viability. LPS-activated BV2-derived CM best recapitulates AD-related pathology, offering a relevant in vitro model for further studies.

## 1. Introduction

Neuroinflammation has emerged as a central contributor to the pathogenesis of neurodegenerative diseases, including Alzheimer’s disease (AD), Parkinson’s disease, and amyotrophic lateral sclerosis [1,2]. Once considered a secondary response to neuronal injury, inflammation is now recognized as an early and active driver of disease progression [2,3]. Both clinical and experimental evidence support the involvement of glial activation and inflammatory cytokine production in synaptic dysfunction, neuronal loss, and cognitive decline [4,5].

The underlying mechanisms of neuroinflammation in neurodegeneration involve complex interactions between activated microglia, astrocytes, and neurons. Activated microglia and infiltrating macrophages release a lot of factors that can injure neurons. Pro-inflammatory cytokines such as TNF-α, IL-1β, and IL-6 can disrupt synaptic function, alter blood–brain barrier permeability, and trigger apoptotic pathways in neurons [6,7]. Furthermore, pathological stimuli such as aggregated amyloid-β (Aβ) peptides, tau tangles, or environmental insults also activate innate immune responses, primarily via Toll-like receptors and the NLRP3 inflammasome pathway [8,9]. This results in the release of pro-inflammatory cytokines (e.g., IL-1β, TNF-α, IL-6), reactive oxygen species (ROS), and chemokines that perpetuate a neurotoxic microenvironment. Neuroinflammation and the Alzheimer’s-related processes thus mutually reinforce each other, leading to exaggerated neurodegeneration [2,10].

Neuroinflammation is intricately linked with several pathological features of neurodegenerative diseases, including oxidative stress, impaired autophagy, mitochondrial and endoplasmic reticulum dysfunction, and apoptosis [6,11]. Prolonged activation of microglia and astrocytes leads to an inflammatory environment, which exacerbates oxidative stress by increasing the production of ROS [6,12]. Elevated ROS levels can damage cellular components, including lipids, proteins, and DNA, thereby compromising neuronal integrity [11].

Mitochondrial dysfunction is a prominent feature in Alzheimer’s disease, characterized by impaired energy metabolism, disrupted calcium homeostasis, and defective mitochondrial dynamics [13,14]. Inflammation-induced elevated ROS level damages mitochondrial DNA, proteins, and lipids, impairing mitochondrial function resulting in decreased ATP production and further ROS generation and creating a vicious cycle that contributes to neuronal death [12,14]. Studies have shown impaired electron transport chain activity and increased oxidative damage in affected brain regions of AD subjects [14]. Additionally, impaired mitophagy—the selective degradation of damaged mitochondria—leads to the accumulation of dysfunctional mitochondria, exacerbating neuronal damage [13].

Although autophagy activation can also protect neurons from inflammatory response by degrading pro-inflammatory factors without affecting the initial immune response, this process is also severely dysregulated in AD [15,16]. Defective autophagy impairs the clearance of misfolded proteins, including amyloid-beta (Aβ) and hyperphosphorylated tau, leading to their accumulation and aggregation and contributing to the formation of amyloid plaques and neurofibrillary tangles, hallmark features of AD pathology [17,18].

Insulin resistance within the brain has garnered significant attention in the context of AD, leading to its characterization as “type 3 diabetes” [19,20]. In the central nervous system, insulin signaling plays a crucial role in regulating neuronal survival, synaptic plasticity, and glucose metabolism. Impaired insulin signaling disrupts these processes, contributing to cognitive decline and neurodegeneration [19,21].

Neuroinflammation is a key contributor to the development of brain insulin resistance [22,23]. Pro-inflammatory cytokines, particularly TNF-α and IL-1β, interfere with insulin receptor signaling by promoting the serine phosphorylation of insulin receptor substrate proteins, thereby inhibiting downstream signaling pathways, such as the PI3K/Akt one [20,24]. This inhibition, in addition to compromising the prosurvival effect of insulin also impairs glucose uptake and metabolism in neurons, leading to energy deficit and increased vulnerability to oxidative stress [21].

Moreover, insulin resistance exacerbates the pathological features of AD by influencing the processing of amyloid precursor protein (APP) and tau phosphorylation. Impaired insulin signaling enhances the activity of enzymes involved in Aβ production and reduces its clearance promoting plaque formation [25]. Additionally, insulin resistance leads to the activation of glycogen synthase kinase-3β (GSK-3β), a key enzyme responsible for tau hyperphosphorylation, contributing to the formation of neurofibrillary tangles [25]. The interplay between insulin resistance and neuroinflammation establishes a detrimental feedback loop, wherein each process exacerbates the other, accelerating neuronal dysfunction and cognitive decline in AD [20].

Despite growing knowledge, current in vitro models of Alzheimer’s disease frequently fall short in replicating these multifactorial aspects. While iPSC-derived neurons and brain organoids provide high physiological relevance, they are technically demanding, expensive, and poorly suited for high-throughput applications [26,27]. On the other hand, more scalable models often lack inflammatory or metabolic components, or rely on simplified readouts that do not capture disease complexity. As a result, there is a pressing need for affordable, reproducible, and modular in vitro models that integrate inflammatory signaling and neuronal vulnerability.

In this study, our aim was to evaluate a simplified neurodegeneration model by characterizing the effects of secreted factors from both unstimulated and LPS-activated inflammatory cell lines on neuron-like cells. We sought to determine whether such a system could provide a cost-effective yet informative platform for high-throughput drug screening, while reflecting key features of neuroinflammation and its downstream processes.

## 2. Results

### 2.1. Effect of Immune Cell-Derived Conditioned Media on Cell Viability

To assess the extent of cell damage and evaluate insulin responsiveness, SH-SY5Y cells were treated with conditioned media derived from various immune cell lines. Insulin responsiveness was represented by concentration-dependent curves, while baseline cytotoxicity levels were visualized using bar charts.

**Figure 1:**
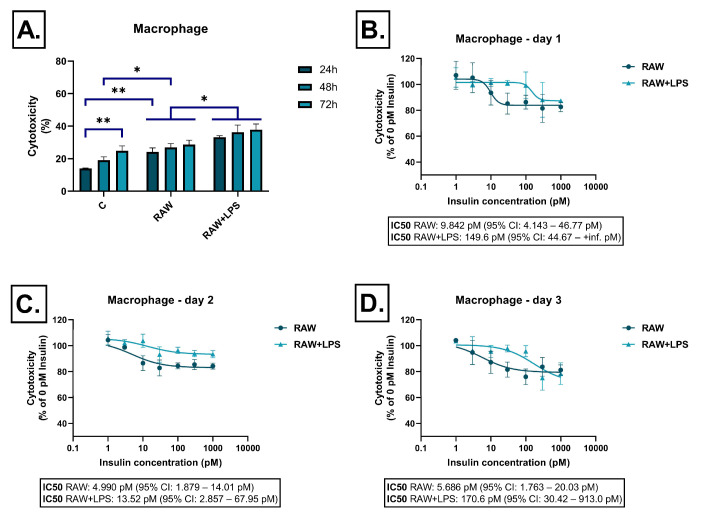
Effect of RAW264.7-derived conditioned media on LDH release in SH-SY5Y cells after 24 h, 48 h, and 72 h of treatment. Cells were exposed to conditioned media derived from RAW264.7 cells, either unstimulated or stimulated with LPS, and treated with insulin (0–1000 pM). LDH release was measured as a marker of cytotoxicity and expressed as a percentage of total cell lysis, normalized to spontaneous and maximal LDH release. Data are presented as % cytotoxicity. Panel (**A**) shows a bar chart representing baseline cytotoxicity levels at 24, 48, and 72 h without insulin treatment. Panels (**B**–**D**) show the effect of insulin (0–1000 pM) on cytotoxicity. In all cases, cytotoxicity is expressed as a percentage relative to the 0 pM insulin condition. C: control; LPS: lipopolysaccharide, * *p* < 0.05; ** *p* < 0.01.

Conditioned medium derived from the RAW264.7 cell line induced moderate cell damage, which slightly increased over time but did not exceed 30% at 72 h. LPS stimulation resulted in significantly higher cytotoxicity at all time points (Figure 1A). Insulin exerted only mild, yet dose-dependent, cytoprotective effects at each time point (Figure 1B–D).

**Figure 2:**
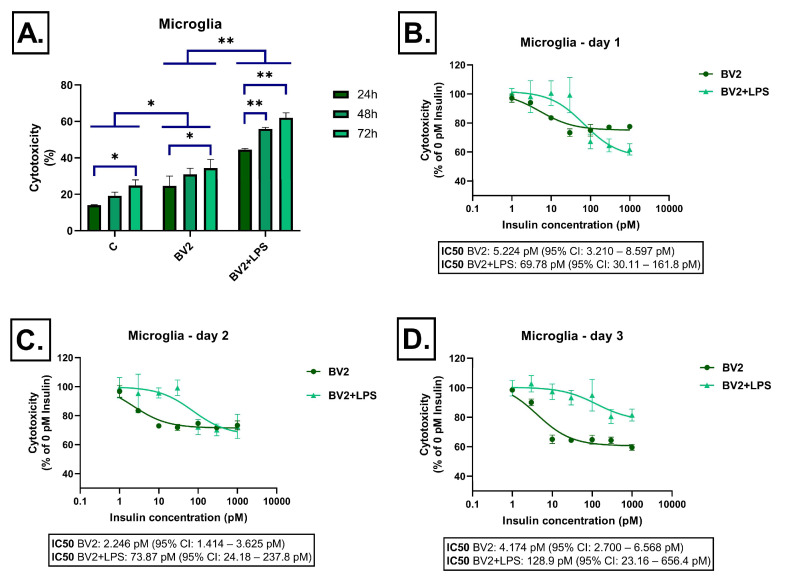
Effect of BV-2-derived conditioned media on LDH release in SH-SY5Y cells after 24 h, 48 h, and 72 h of treatment. Cells were exposed to conditioned media derived from BV-2 cells, either unstimulated or stimulated with LPS, and treated with insulin (0–1000 pM). LDH release was measured as a marker of cytotoxicity and expressed as a percentage of total cell lysis, normalized to spontaneous and maximal LDH release. Data are presented as % cytotoxicity. Panel (**A**) shows a bar chart representing baseline cytotoxicity levels at 24, 48, and 72 h without insulin treatment. Panels (**B**–**D**) show the effect of insulin (0–1000 pM) on cytotoxicity. In all cases, cytotoxicity is expressed as a percentage relative to the 0 pM insulin condition. C: control; LPS: lipopolysaccharide, * *p* < 0.05; ** *p* < 0.01.

Conditioned medium from BV2 microglial cells without LPS stimulation caused moderate cytotoxicity, which gradually increased over time. In the presence of LPS, cytotoxicity was significantly higher at all time points, exceeding 60% at 72 h (Figure 2A). Upon insulin treatment, consistent and concentration-dependent reductions in cytotoxicity were observed in the LPS-free BV2 groups at all time points. In contrast, in the LPS-stimulated BV2 groups, the effectiveness of insulin decreased over time, and by 72 h, only minimal reductions in cytotoxicity were observed even at the highest insulin concentrations (Figure 2B–D).

**Figure 3:**
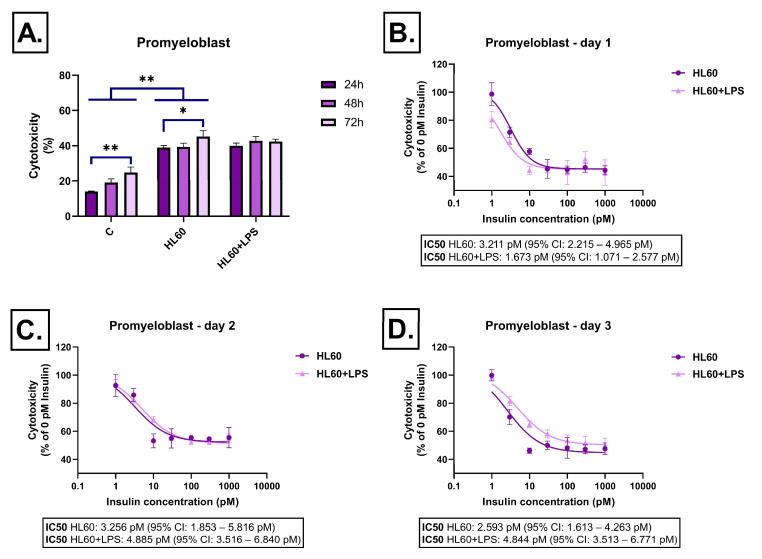
Effect of HL-60-derived conditioned media on LDH release in SH-SY5Y cells after 24 h, 48 h, and 72 h of treatment. Cells were exposed to conditioned media derived from HL-60 cells, either unstimulated or stimulated with LPS, and treated with insulin (0–1000 pM). LDH release was measured as a marker of cytotoxicity and expressed as a percentage of total cell lysis, normalized to spontaneous and maximal LDH release. Data are presented as % cytotoxicity. Panel (**A**) shows a bar chart representing baseline cytotoxicity levels at 24, 48, and 72 h without insulin treatment. Panels (**B**–**D**) show the effect of insulin (0–1000 pM) on cytotoxicity. In all cases, cytotoxicity is expressed as a percentage relative to the 0 pM insulin condition. C: control; LPS: lipopolysaccharide, * *p* < 0.05; ** *p* < 0.01.

In the case of undifferentiated HL-60 cells, the presence of LPS did not significantly increase cytotoxicity (Figure 3A). Insulin treatment reduced cytotoxicity starting from 10 pM, with more pronounced effects at higher concentrations. This pattern remained consistent throughout the three-day treatment period (Figure 3B–D).

**Figure 4:**
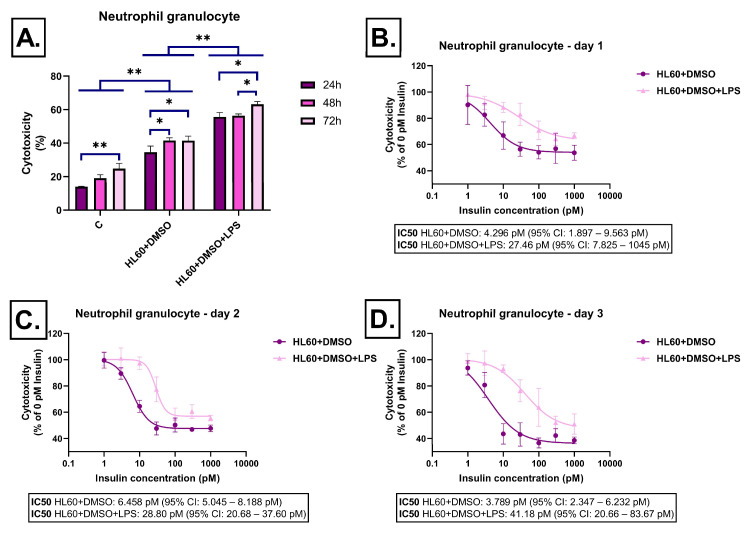
Effect of dimethyl sulfoxide-differentiated HL-60-derived conditioned media on LDH release in SH-SY5Y cells after 24 h, 48 h, and 72 h of treatment. Cells were exposed to conditioned media derived from dimethyl sulfoxide-differentiated HL-60 cells, either unstimulated or stimulated with LPS, and treated with insulin (0–1000 pM). LDH release was measured as a marker of cytotoxicity and expressed as a percentage of total cell lysis, normalized to spontaneous and maximal LDH release. Data are presented as % cytotoxicity. Panel (**A**) shows a bar chart representing baseline cytotoxicity levels at 24, 48, and 72 h without insulin treatment. Panels (**B**–**D**) show the effect of insulin (0–1000 pM) on cytotoxicity. In all cases, cytotoxicity is expressed as a percentage relative to the 0 pM insulin condition. C: control; LPS: lipopolysaccharide, DMSO: dimethyl-sulfoxide, * *p* < 0.05; ** *p* < 0.01.

In DMSO-differentiated HL-60 cells, LPS-stimulated conditioned medium induced significantly higher cytotoxicity, particularly on the third day, when levels exceeded 60% (Figure 4A). Insulin exerted protective effects already at low concentrations, and these effects became stronger over time. By the third day, cytotoxicity levels were significantly reduced, especially at medium and high insulin concentrations, indicating time-dependent, insulin-mediated protection (Figure 4B–D).

**Figure 5:**
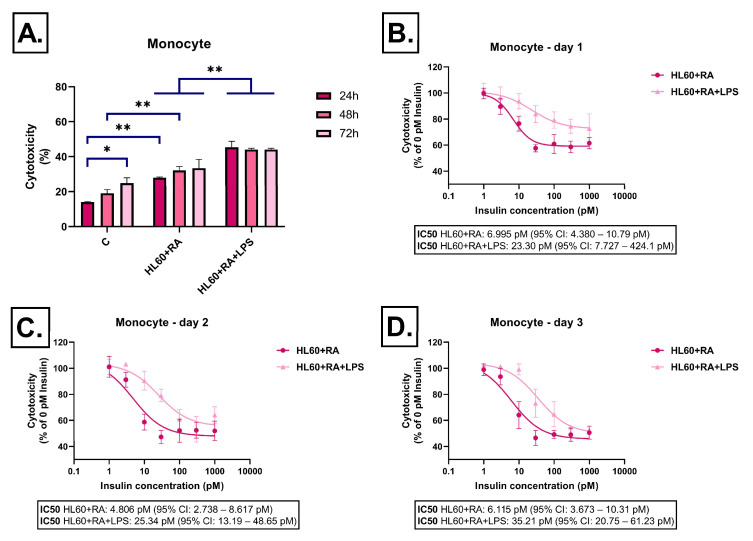
Effect of all-trans retinoic acid-differentiated HL-60-derived conditioned media on LDH release in SH-SY5Y cells after 24 h, 48 h, and 72 h of treatment. Cells were exposed to conditioned media derived from all-trans retinoic acid-differentiated HL-60 cells, either unstimulated or stimulated with LPS, and treated with insulin (0–1000 pM). LDH release was measured as a marker of cytotoxicity and expressed as a percentage of total cell lysis, normalized to spontaneous and maximal LDH release. Data are presented as % cytotoxicity. Panel (**A**) shows a bar chart representing baseline cytotoxicity levels at 24, 48, and 72 h without insulin treatment. Panels (**B**–**D**) show the effect of insulin (0–1000 pM) on cytotoxicity. In all cases, cytotoxicity is expressed as a percentage relative to the 0 pM insulin condition. C: control; LPS: lipopolysaccharide, RA: all-trans retinoic acid, * *p* < 0.05; ** *p* < 0.01.

RA-differentiated HL-60 cells displayed a similar pattern to the DMSO-differentiated group. Cell damage remained moderate but was increased by LPS stimulation (Figure 5A). Insulin reduced cytotoxicity starting from 10 pM, and this protective effect became more pronounced by the third day (Figure 5B–D).

### 2.2. Effect of Conditioned Media on ROS Production

Alterations in ROS levels can provide a valuable insight into the effects of immune cell-derived inflammatory factors on neurons. For this purpose, SH-SY5Y cells were treated with conditioned media from various immune cell types for 3 h or 3 days, and hydrogen peroxide and superoxide levels were assessed.

**Figure 6:**
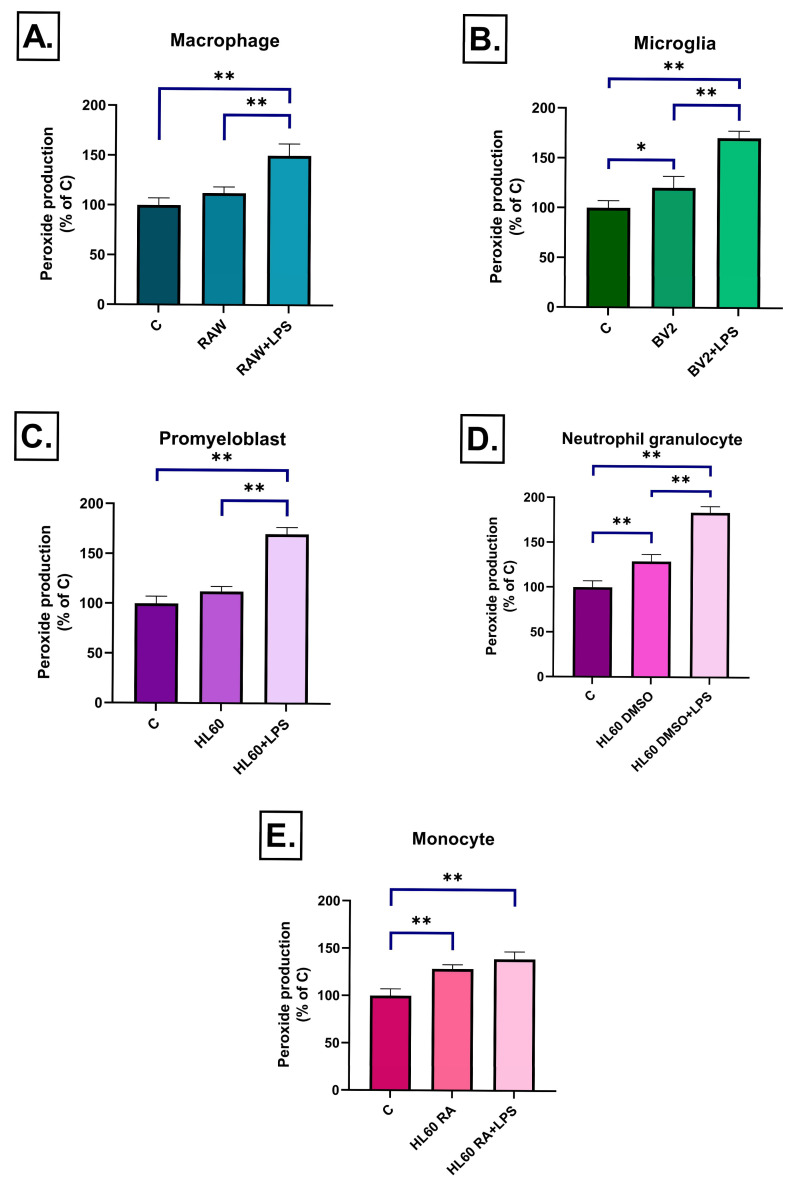
Peroxide production in SH-SY5Y cells after 3 h of treatment with conditioned media from various immune cell types (**A**–**E**), measured by 2′,7′-dichlorofluorescin diacetate (DCFDA) fluorescence. LPS-stimulated conditioned media induced a significant increase in ROS levels compared to the control in all cell line groups. Data are expressed as a percentage of the control. C: control, LPS: lipopolysaccharide, RA: all-trans retinoic acid, DMSO: dimethyl sulfoxide, * *p* < 0.05; ** *p* < 0.01.

**Figure 7:**
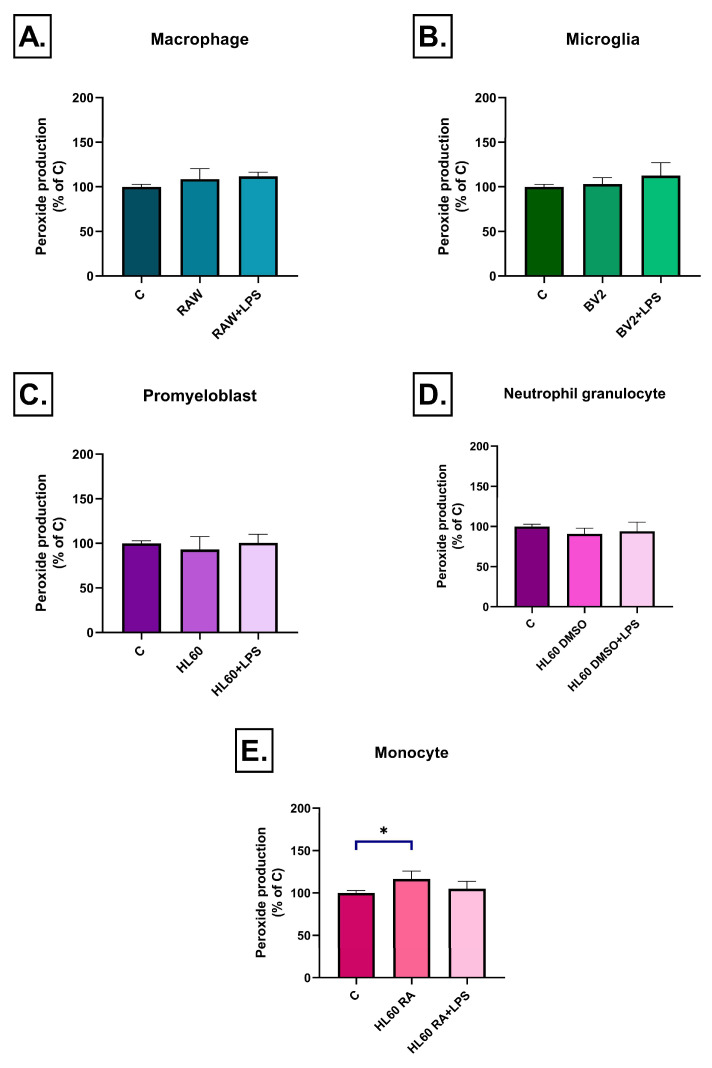
Peroxide production in SH-SY5Y cells after 72 h of treatment with conditioned media from various immune cell types (**A**–**E**), measured by 2′,7′-dichlorofluorescin diacetate (DCFDA) fluorescence. Compared to the 3 h time point, peroxide levels were normalized by 72 h in most groups, shown by no significant differences relative to the control, with the exception of the RA-differentiated HL60 cell medium. Data are expressed as a percentage of the control. C: control, LPS: lipopolysaccharide, RA: all-trans retinoic acid, DMSO: dimethyl sulfoxide, * *p* < 0.05.

After 3 h of exposure, almost all treatments increased peroxide production, with the most pronounced responses observed in the DMSO- and RA-differentiated HL60 medium-treated groups, both with and without LPS stimulation (Figure 6D,E). BV2-derived medium significantly elevated peroxide levels even in the absence of LPS, and this effect was further enhanced by LPS treatment (Figure 6B). In contrast, RAW264.7 and undifferentiated HL-60 cell media induced peroxide production only following LPS stimulation (Figure 6A,C). By day 3, peroxide levels had normalized in most groups (Figure 7A–D), except for RA-differentiated HL60-derived medium-treated ones, which still showed a slight but significant increase compared to the control (Figure 7E).

**Figure 8:**
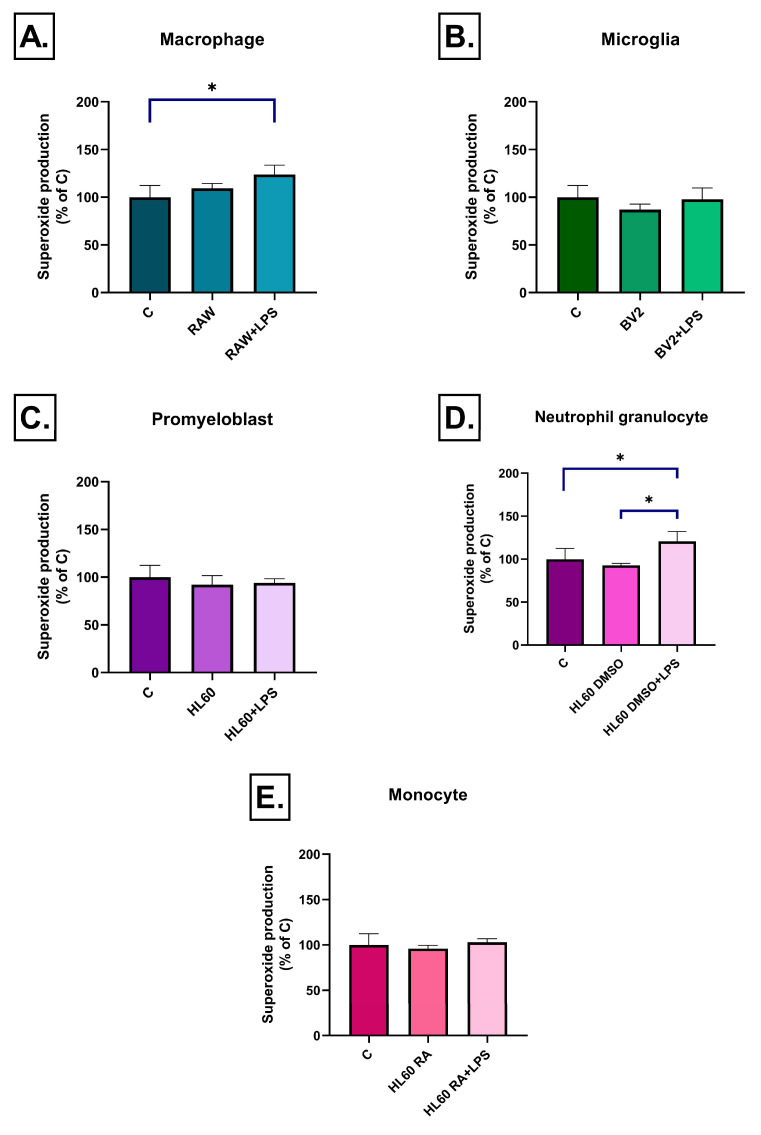
Superoxide production in SH-SY5Y cells after 3 h of treatment with conditioned media from various immune cell types (**A**–**E**), measured by dihydroethidium (HE) fluorescence. A significant increase in superoxide production was observed only in response to the LPS-stimulated conditioned media of RAW264.7 and DMSO-differentiated HL60 cells, while no significant changes were detected in the other groups. Data are expressed as a percentage of the control. C: control, LPS: lipopolysaccharide, RA: all-trans retinoic acid, DMSO: dimethyl sulfoxide, * *p* < 0.05.

**Figure 9:**
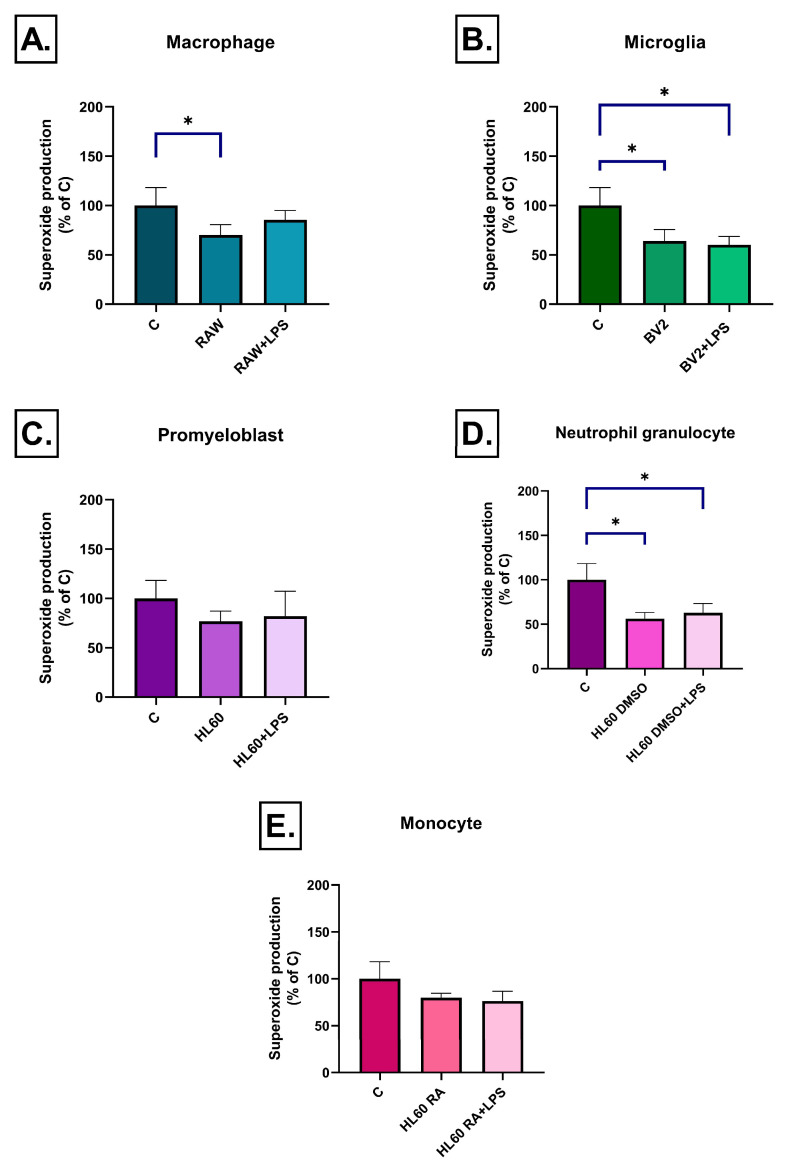
Superoxide production in SH-SY5Y cells after 72 h of treatment with conditioned media from various immune cell types (**A**–**E**), measured by dihydroethidium (HE) fluorescence. A significant decrease in superoxide production was observed in response to LPS-stimulated conditioned media from RAW264.7, BV2, and DMSO-differentiated HL-60 cells, while no significant changes were detected in the other two groups. Data are expressed as a percentage of the control. C: control, LPS: lipopolysaccharide, RA: all-trans retinoic acid, DMSO: dimethyl sulfoxide, * *p* < 0.05.

Superoxide production exhibited a more complex, cell-type and time-dependent pattern. After 3 h, RAW264.7 cell medium induced increased superoxide levels only after LPS stimulation, while RAW264.7 medium without LPS treatment had no effect (Figure 8A). Interestingly, by day 3, superoxide levels were significantly reduced in the unstimulated RAW264.7 medium treated group compared to the control one, suggesting a compensatory or adaptive response (Figure 9A). BV2 cell medium induced no early effect on superoxide production (Figure 8B), but after 3 days, in both the LPS-treated and untreated groups a moderate but significant decrease was detected (Figure 9B). Among the HL-60 groups, only DMSO-differentiated HL60 cell medium triggered superoxide accumulation after LPS stimulation at 3 h (Figure 8D). By day 3, media from these cells—both LPS-treated and untreated—led to a reduction in superoxide levels (Figure 9D). No significant changes in superoxide production were detected in either undifferentiated or RA-differentiated HL-60 groups at any time point (Figure 8C,E and Figure 9C,E).

### 2.3. Effect of Inflammatory Conditioned Media on Mitochondrial Membrane Potential

Mitochondrial membrane potential is a key indicator of cellular energy production and viability, and its reduction reflects mitochondrial dysfunction, which contributes to the development of neurodegenerative diseases. To assess the effects of conditioned media derived from activated immune cells on SH-SY5Y cells, changes in mitochondrial membrane potential were measured using JC-1 staining.

**Figure 10:**
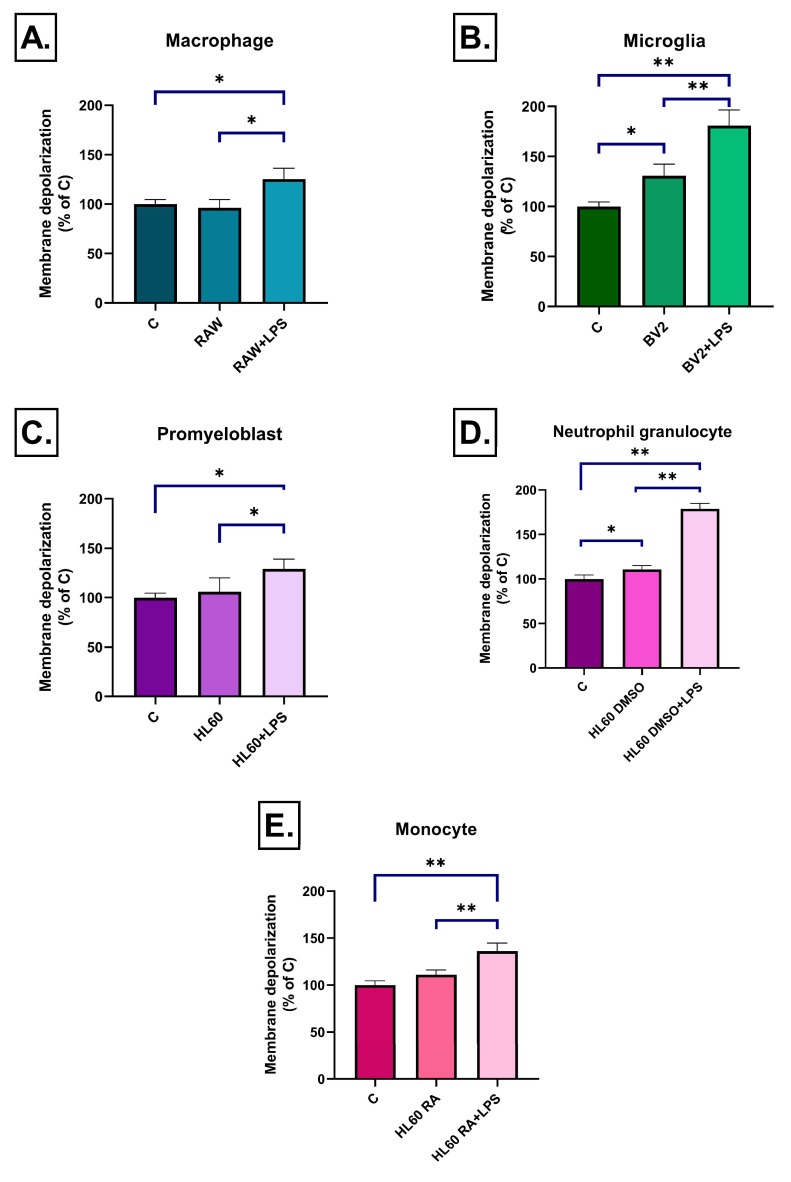
Mitochondrial membrane potential in SH-SY5Y cells after 3 h of treatment with conditioned media from various immune cell types (**A**–**E**), measured by JC-1 fluorescence. A significant increase in the green/red fluorescence ratio was observed in all LPS-treated groups, indicating mitochondrial depolarization compared to the control. Data are expressed as a percentage of the control. C: control, LPS: lipopolysaccharide, RA: all-trans retinoic acid, DMSO: dimethyl sulfoxide, * *p* < 0.05; ** *p* < 0.01.

After a short incubation period (3 h), conditioned media from BV2 microglial cells induced the strongest mitochondrial depolarization among the tested cell lines. Unstimulated BV2 medium caused a slight but significant reduction in the mitochondrial membrane potential, which was strongly enhanced in the LPS stimulated group (Figure 10B). For the case of conditioned media from RAW264.7 macrophages a mild but significant depolarization was seen only after LPS stimulation (Figure 10A).

For the media of HL-60 cells, the state of differentiation had a notable impact on mitochondrial membrane potential changes. Media of undifferentiated HL60 cells induced a moderate depolarization only upon LPS treatment (Figure 10C). In contrast, DMSO-differentiated HL60 medium-treated groups exhibited a significant depolarizing effect even in the absence of LPS, which was further intensified by LPS treatment (Figure 10D). RA-differentiated HL60 cell medium induced significant mitochondrial membrane depolarization only after LPS stimulation (Figure 10E).

**Figure 11:**
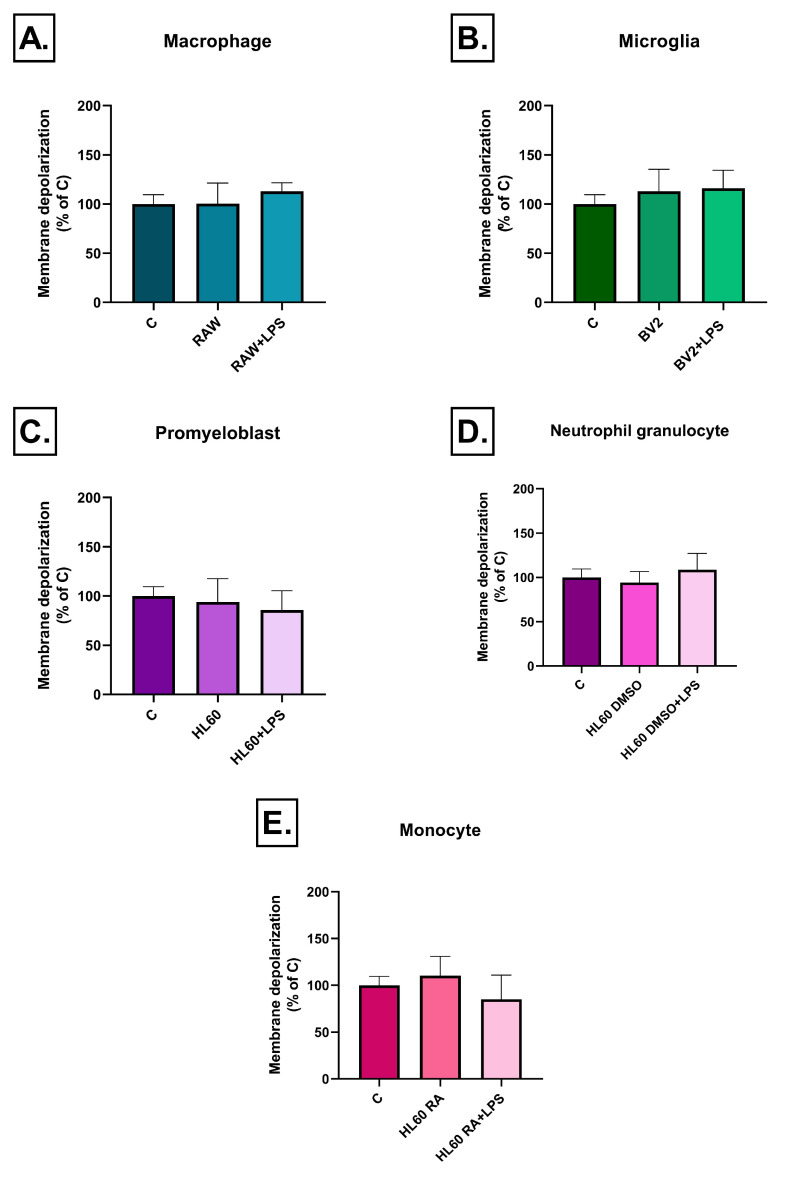
Mitochondrial membrane potential in SH-SY5Y cells after 72 h of treatment with conditioned media from various immune cell types (**A**–**E**), measured by JC-1 fluorescence. Compared to the 3-h time point, the green/red fluorescence ratio returned to baseline levels in all groups by 72 h, with no significant differences relative to the control. Data are expressed as a percentage of the control. C: control, LPS: lipopolysaccharide, RA: all-trans retinoic acid, DMSO: dimethyl sulfoxide.

Notably, after prolonged exposure (3 days), mitochondrial membrane depolarization effects were no longer detectable in response to any of the conditioned media treatments, suggesting a transient mitochondrial dysfunction due to inflammatory stress (Figure 11).

### 2.4. Effect of Inflammatory Conditioned Media on Mitochondrial Mass

Mitochondrial mass is an important indicator of cellular metabolism and functional status, and its reduction is often associated with mitochondrial dysfunction, particularly under neurodegenerative and inflammatory conditions. The extent and alterations of the mitochondrial network can be quantitatively assessed using MitoTracker staining. Accordingly, SH-SY5Y cells were treated with conditioned media derived from various immune cell types, supplemented with LPS and insulin, and mitochondrial mass was determined.

**Figure 12:**
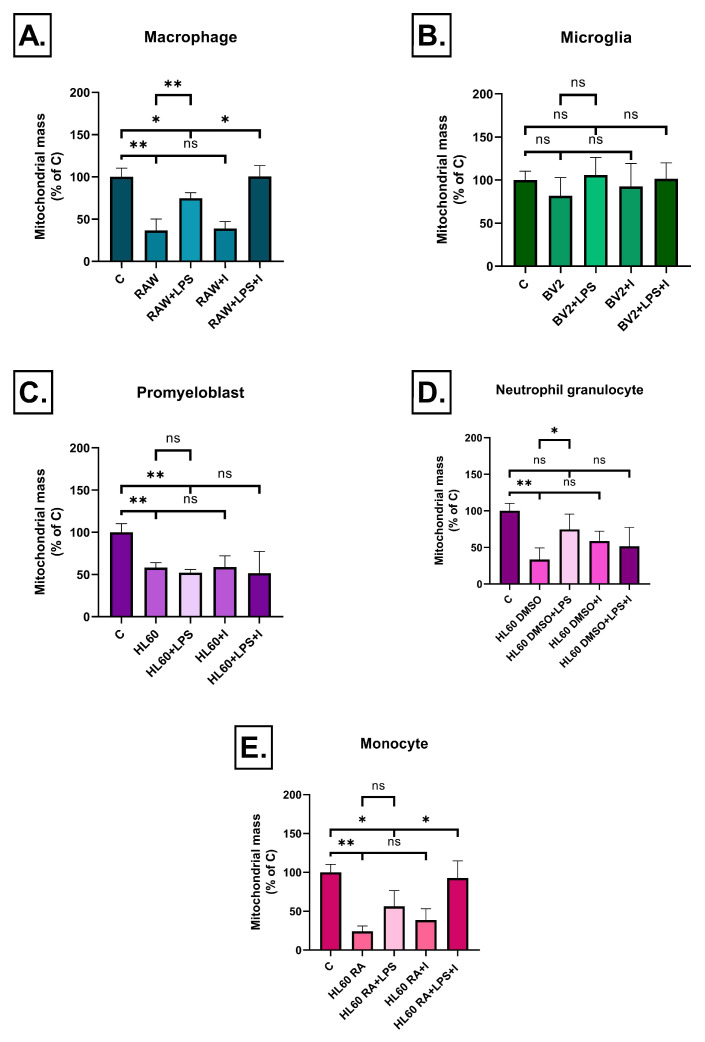
Mitochondrial mass in SH-SY5Y cells after 72 h of treatment with conditioned media from various immune cell types (**A**–**E**), measured by MitoTracker fluorescence. Changes in mitochondrial signal intensity varied between treatment groups. Data are expressed as a percentage of the control. C: control, LPS: lipopolysaccharide, RA: all-trans retinoic acid, DMSO: dimethyl sulfoxide, * *p* < 0.05; ** *p* < 0.01, ns: not significant.

RAW264.7 cell medium treatment resulted in a significant decrease in the mitochondrial mass of SH-SY5Y cells compared to the control both in the absence and presence of LPS. Interestingly, compared to untreated RAW264.7 cells, their LPS treatment led to a significant increase in mitochondrial mass. The introduction of insulin significantly restored the mitochondrial mass in the presence of LPS. There was no significant impact of insulin on the effect of untreated RAW264.7 medium (Figure 12A).

In contrast, in the case of BV2 microglial cells, none of the treatment conditions resulted in a significant change in mitochondrial mass (Figure 12B).

In case of undifferentiated HL60 promyeloblast cells, the conditioned media of both untreated and LPS-treated cells significantly reduced the mitochondrial mass of SH-SY5Y cells compared to the control. However, insulin treatment had no significant effect (Figure 12C).

In case of DMSO-differentiated HL-60 cells, the conditioned medium of untreated cells caused a strong reduction in mitochondrial mass compared to the control. Notably, LPS treatment of DMSO-differentiated HL60 cells resulted in a higher mitochondrial mass compared to the untreated ones and there was no significant difference between the control and HL60 + DMSO + LPS groups. Insulin treatment in this group also did not result in significant changes (Figure 12D).

The media of RA-differentiated HL60 cells caused a significant decrease in mitochondrial mass of SH-SY5Y cells compared to the control both in the untreated and LPS-treated groups. Insulin treatment restored the mitochondrial mass in the LPS-induced group but showed no significant effect in the untreated group (Figure 12E).

### 2.5. Effect of Inflammatory Conditioned Media on Glucose Uptake

**Figure 13:**
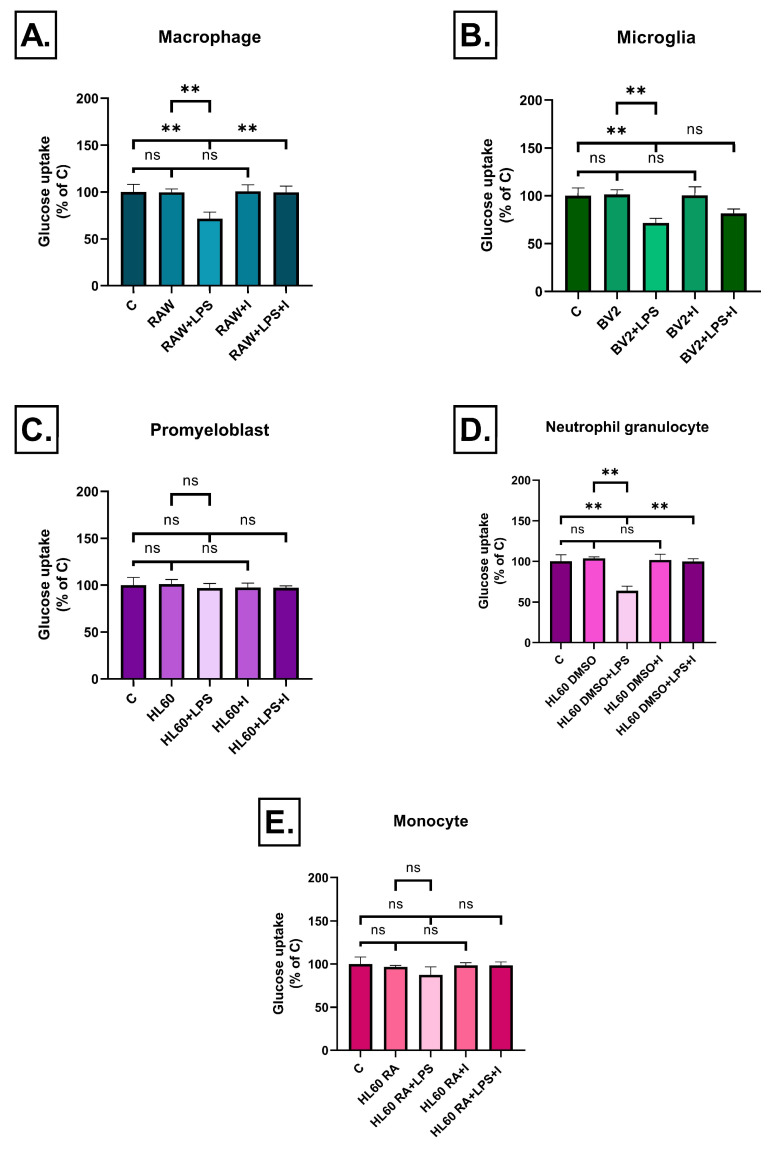
Glucose uptake into SH-SY5Y cells after 72 h of treatment with conditioned media from various immune cell types (**A**–**E**), measured by 2-NBDG fluorescence. Reduced glucose uptake was seen in the case of LPS-treated RAW264.7, BV2 and DMSO differentiated HL60 cells. Insulin treatment reversed it only in the case of RAW264.7 cells. Data are expressed as a percentage of the control. C: control, LPS: lipopolysaccharide, RA: all-trans retinoic acid, DMSO: dimethyl sulfoxide, ** *p* < 0.01, ns: not significant.

Glucose uptake in neuronal cells is essential for maintaining normal cellular function and energy supply, especially in the highly energy-demanding environment of the brain. Impaired glucose uptake and insulin resistance play a central role in the pathogenesis of several neurodegenerative diseases, including Alzheimer’s disease. Inflammatory mediators may negatively affect glucose transport, contributing to metabolic dysfunction in neurons. Accordingly, we assessed the effects of various immune cell-derived conditioned media on glucose uptake into SH-SY5Y cells. No change in glucose uptake was seen when conditioned media of untreated cells were used either alone or in combination with insulin. In the case of LPS treated cells, a different effect was detected depending on cell type. In case of treatments with conditioned media from LPS-stimulated undifferentiated or RA-differentiated HL60 cells, neither the medium nor insulin treatment resulted in significant changes in glucose uptake (Figure 13C,E). The conditioned medium of LPS-treated RAW264.7 macrophages and DMSO-differentiated HL60 granulocytes significantly reduced glucose uptake into SH-SY5Y cells. Insulin treatment, however, completely restored their effect (Figure 13A,D).

In the case of BV2 microglial cells the conditioned medium of LPS-treated cells significantly impaired glucose uptake. However, contrary to RAW264.7 and HL60 neutrophils, the found reduction was not reversed by insulin treatment (Figure 13B).

### 2.6. Effect of Immune Cell-Conditioned Media on Autophagic Activity

Autophagy is a cellular survival mechanism that enables the degradation and recycling of damaged organelles and proteins, especially under stress or injury. The dysregulation of autophagy contributes to the accumulation of pathological protein aggregates and neuronal loss in neurodegenerative diseases. Inflammatory conditions can significantly influence autophagic activity in neuronal cells. Therefore, we evaluated the effects of immune cell-derived conditioned media on autophagic activity in SH-SY5Y cells.

**Figure 14:**
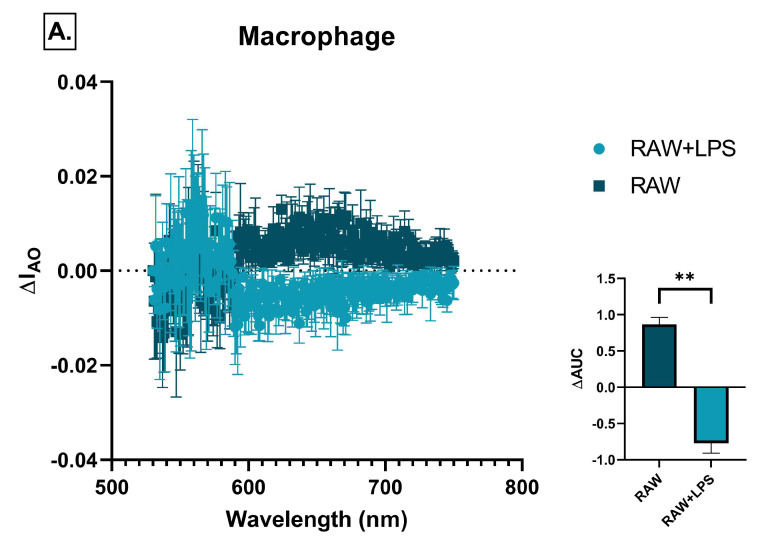

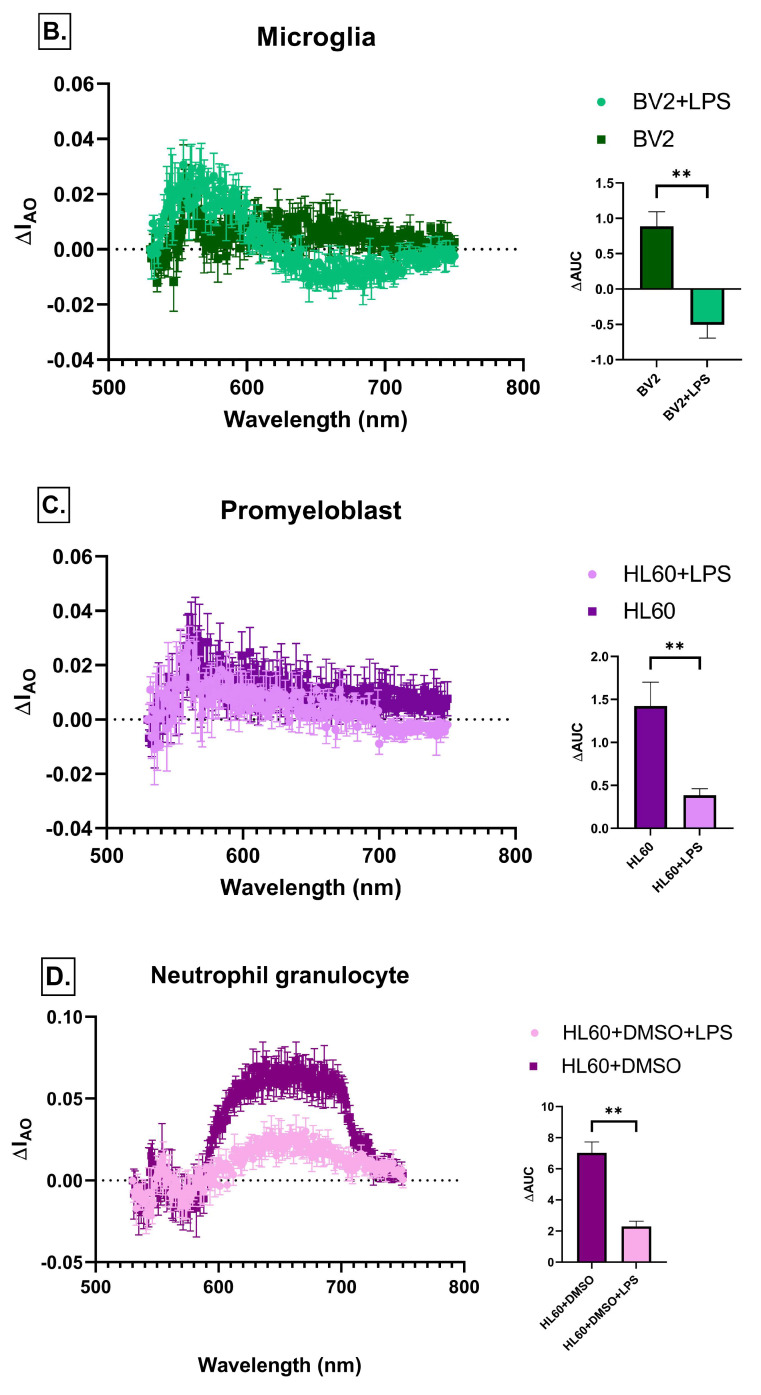

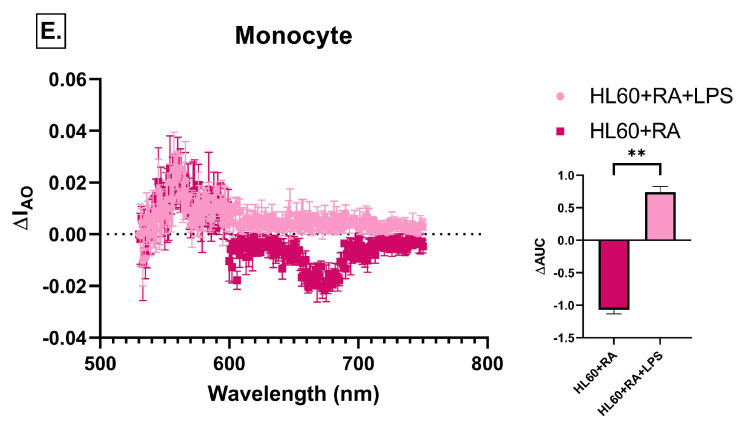
Autophagic vacuole accumulation in SH-SY5Y cells after 72 h of treatment with conditioned media from various immune cell types (**A**–**E**), measured by acridine orange staining. Fluorescence data were normalized to cell number, and control values were subtracted from each treatment group. Representative spectra are shown alongside bar graphs of the corresponding area under the curve (ΔAUC) values. C: control, LPS: lipopolysaccharide, RA: all-trans retinoic acid, DMSO: dimethyl sulfoxide, ** *p* < 0.01.

A significant decrease in acridine orange fluorescence was observed in SH-SY5Y cells in response to LPS-treated RAW264.7 and BV2 conditioned media compared to untreated ones, indicating the suppression of autophagic activity under inflammatory conditions. The magnitude of the effect was similar in the two cell types (Figure 14A,B).

Similarly, a significant decrease was also observed in the case of LPS-treated HL60 cells, although the extent of the decrease was less pronounced compared to that seen with RAW264.7 or BV2 cells media (Figure 14C).

DMSO-differentiated HL60 cells induced high baseline acridine orange fluorescence, which may indicate elevated basal levels of acidic organelles and autophagic activity. Following LPS-stimulation, a significant decrease in fluorescence was observed, which again suggests a reduced autophagic activity under inflammatory conditions (Figure 14D).

In contrast, RA-differentiated HL60 cells decreased the baseline acridine orange fluorescence, possibly reflecting reduced basal autophagy. Following LPS treatment, fluorescence levels increased in this group, which may indicate partial restoration or activation of autophagic processes in this monocyte-like cell population (Figure 14E).

### 2.7. Effect of Immune Cell-Conditioned Media on Insulin Signaling

GSK-3β plays a key role in the phosphorylation of tau protein, thereby linking insulin signaling to the formation of neurofibrillary tangles (NFTs). Since GSK-3β inactivation by its insulin-induced phosphorylation can inhibit excessive tau phosphorylation, we analyzed it to study the relationship between insulin resistance and tau pathology.

According to the above results, BV2 cells were found to be the most promising for inducing insulin resistance-related neuronal damage. We measured pGSK3β levels in SH-SY5Y cells treated with its conditioned medium after stimulation with various insulin concentrations (10–1000 pM). In the control group, pGSK3β levels increased proportionally with the insulin dose, indicating an intact insulin signaling response. The conditioned medium of untreated BV2 cells did not cause significant changes compared to the control.

In contrast, in the case of LPStreated BV2 cells, conditioned medium resulted in decreased basal pGSK3β level and an impaired response to insulin, although maximal phosphorylation was still achieved at high insulin concentration. This suggests that inflammation induced insulin resistance, characterized by its reduced potency with unchanged efficacy.

**Figure 15:**
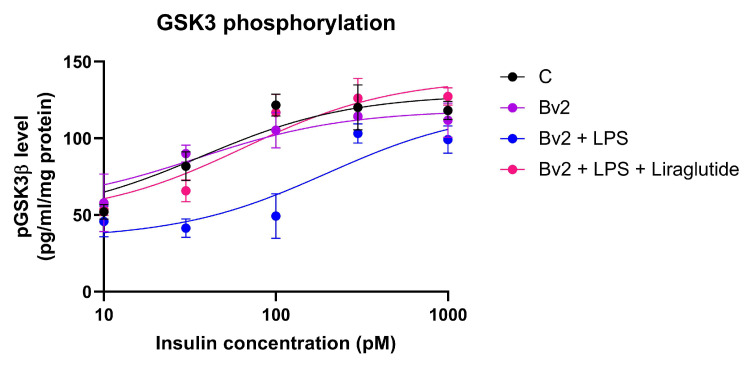
Phosphorylation of GSK3β in SH-SY5Y cells after 72 h of treatment with conditioned media derived from BV2 microglia, with or without liraglutide, followed by insulin stimulation (1 h, 1–1000 pM). C: control, LPS: lipopolysaccharide.

When used in combination with liraglutide, a GLP-1 analog with known insulin-sensitizing effects, insulin-induced pGSK3β levels were restored to control values, indicating reversibility of inflammation-induced insulin resistance (Figure 15).

### 2.8. Effect of Immune Cell-Conditioned Media on Protein Aggregation

Abnormal protein aggregation, particularly the accumulation of β-sheet-rich structures, plays a central role in the pathogenesis of neurodegenerative diseases such as Alzheimer’s disease. Inflammatory mediators may accelerate this process by disrupting protein homeostasis and impairing aggregate clearance. We assessed the influence of BV2 cell-derived conditioned media on the extent of protein aggregation in SH-SY5Y cells by thioflavin S staining.

**Figure 16:**
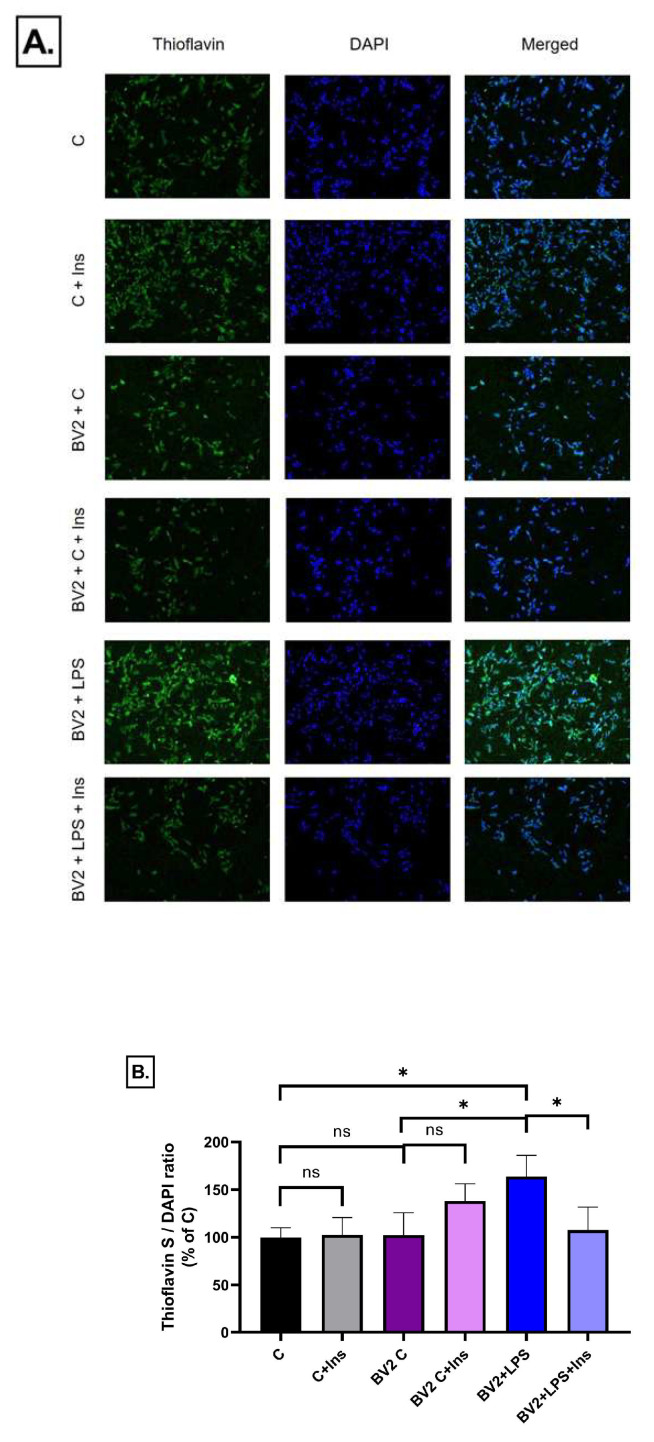
Thioflavin S staining of protein aggregates in SH-SY5Y cells after treatment with conditioned media from BV2 microglia, with or without LPS and/or insulin (1000 pM) treatment. (**A**) Representative fluorescence images showing thioflavin S (green), DAPI (blue), and merged channels. (**B**) Quantification of protein aggregation expressed as the ratio of thioflavin S to DAPI fluorescence. Conditioned media from LPS-stimulated BV2 cells induced a significant increase in amyloid aggregation that was reversed by insulin treatment. C: control, LPS: lipopolysaccharide, I: insulin, * *p* < 0.05; ns: not significant.

Based on our results, the conditioned medium of LPS-treated BV2 cells significantly increased protein aggregation compared to the control. Insulin treatment effectively reversed the found accumulation of protein aggregates. On the other hand, no significant alterations were observed in response to untreated BV2 cells, insulin or their combination, indicating that these treatments did not meaningfully affect the extent of protein aggregation (Figure 16).

## 3. Discussion

Neuroinflammatory processes play a central role in the pathophysiology of many neurodegenerative disorders, including Alzheimer’s disease (AD) [1]. Therefore, developing robust, cost-effective, and reproducible in vitro models that recapitulate inflammation-driven neuronal dysfunction is essential for advancing therapeutic discovery [2,28]. In this study, we evaluated how soluble factors secreted by LPS-stimulated immune cell lines affect neuron-like cells, aiming to identify a simple but biologically relevant system that could serve as a platform for high-throughput drug screening.

Despite being a transformed cell line, SH-SY5Y cells remain a widely accepted alternative to primary neurons due to their stability and accessibility in screening studies [29]. To model the immune side of neuroinflammation, various immortalized cell lines are used as substitutes for primary microglia or blood-derived myeloid cells. The BV2 cell line is one of the most commonly used murine microglia immortalized with the raf/myc oncogene. BV2 cells share many properties with primary microglia and mount vigorous inflammatory responses to stimuli, making them a standard in vitro model of microglial activation. Conditioned media from LPS-activated BV2 microglia induced the most complex and sustained neurodegenerative phenotype in SH-SY5Y cells. We observed a progressive reduction in neuronal cell viability, with significant resistance to the cytoprotective effect of insulin. Initial ROS generation followed by sustained superoxide production, early mitochondrial damage, altered autophagy, and impaired glucose uptake are consistent with the neuroinflammatory processes and insulin resistance. The accumulation of protein aggregates and the reversible increase in GSK3β activity further indicate the presence of AD-like pathology. As the elevated GSK3β activity could be restored by liraglutide treatment the reversibility of insulin resistance was also confirmed. These effects are consistent with prior studies showing that BV2-secreted TNF-α and IL-1β induce oxidative stress and mitochondrial dysfunction in neurons, reduce GSH:GSSG ratios, and impair autophagy with the accumulation of p62 and the inhibition of LC3B-II turnover. Levels of apoptosis markers such as cleaved PARP and pro-apoptotic Bax, were elevated, while Bcl-2 decreased—mirroring microglia-induced apoptotic cascades described previously [30,31,32].

Another in vitro inflammation model uses RAW264.7, a mouse monocyte-macrophage cell line. RAW264.7 cells, when treated with LPS or interferons, produce high levels of nitric oxide and cytokines, and thus serve as a convenient proxy for peripheral macrophages or activated microglia in inflammatory assays. Indeed, a systematic review found that the RAW264.7 model is widely employed to screen anti-inflammatory compounds by measuring LPS-induced TNF-α, IL-1β, IL-6, and NO production [33,34,35,36]. RAW264.7 macrophage-conditioned media also induced damage and insulin resistance in SH-SY5Y cells, though the latter was significantly milder than that seen in the BV2 model and could be rescued with insulin. ROS and superoxide levels were elevated early and remained sustained, accompanied by mitochondrial injury and a decrease in mitochondrial number, which normalized upon insulin treatment. Glucose uptake declined transiently but was reversible. Autophagy disruption was observed, though less pronounced than in the case of BV2 cells. Other studies using RAW264.7 cells report lower basal cytokine secretion compared to microglia, with moderate neurotoxicity only after polarization into an M1 phenotype [35,37]. In our setup, LPS activation may not have fully shifted RAW264.7 cells into a strongly pro-inflammatory state; thus, the observed effects likely represent a mixed profile of moderate inflammation and partial neuroprotection.

In addition to microglial and macrophage models, researchers sometimes utilize promyelocytic leukemia cells, like HL60, to study myeloid differentiation and function relevant to neuroinflammation. HL60 cells are human myeloid precursors that can be driven to different mature innate immune cell phenotypes [38,39]. Notably, treating HL60 cells with 1.25% dimethyl sulfoxide (DMSO) for about 5–7 days induces differentiation into neutrophil granulocyte-like cells, characterized by the upregulation of granulocyte markers and capacity for oxidative burst. On the other hand, all-trans retinoic acid (RA) will trigger their differentiation along the monocyte/macrophage lineage, yielding cells that adhere, phagocytose, and secrete inflammatory cytokines. Thus, by choosing the appropriate differentiation agent, HL60 cells provide a flexible model to generate neutrophil-like or monocyte-like cells for research. In neuroinflammation studies, they can serve as a source of human myeloid cells to test how peripheral immune cells might interact with neurons or glia [39,40].

Monocyte-like RA-differentiated HL60 cells produced a distinct inflammatory profile. While they induced sustained ROS production in SH-SY5Y cells, superoxide levels did not increase. Mitochondrial injury occurred early but was reversible with insulin and was accompanied by an initial decrease in mitochondrial numbers that was recovered later. Insulin resistance developed, though glucose uptake remained unaffected, indicating a disconnect between receptor signaling and metabolic function. Autophagy was also impaired. These results suggest that monocyte-like cells can elicit metabolic dysfunction without overt cytotoxicity, a phenomenon aligned with mild-to-moderate inflammatory responses observed in peripheral monocyte infiltration models. Unlike microglia or macrophages, these monocyte-like cells appear to trigger limited secondary neuronal damage [40,41].

Neutrophil-like DMSO-differentiated HL60 cells elicited severe cytotoxicity and strong insulin resistance. We observed high levels of ROS and initial mitochondrial damage, alongside a drop in mitochondrial count and glucose uptake—both of which were reversible with insulin. Autophagic impairment was evident, with features of lysosomal dysfunction and substrate accumulation. This aggressive phenotype suggests that neutrophil-like cells, when activated, may exert acute oxidative stress and metabolic damage, consistent with the literature showing that neutrophil-derived elastases, ROS bursts, and extracellular traps contribute to neurotoxicity in early neurodegenerative lesions [42,43]. However, these cells are less commonly modeled in vitro due to their short lifespan and technical instability.

Undifferentiated HL60 promyeloblasts had a minimal impact on neuronal physiology. SH-SY5Y cells exposed to their conditioned media showed only a mild and transient increase in ROS generation and slight mitochondrial damage. No significant insulin resistance, glucose uptake alteration, mitochondrial number change, or autophagic failure were detected. These results suggest that without differentiation, HL60 cells do not secrete sufficient proinflammatory factors to mimic a neuroinflammatory environment. In line with this, previous studies described undifferentiated HL60 cells as poor cytokine secretors in response to LPS unless primed with other agents, limiting their utility as inflammatory inducers in neurodegeneration models [39,40].

Our data support the feasibility of a sequentially activated system—LPS stimulation of immune cells followed by treatment of neuron-like cells with conditioned medium—as a practical, scalable, and cost-efficient neuroinflammatory model. Compared to more complex systems such as iPSC-derived neurons or cerebral organoids, which are often costly and require advanced infrastructure, this approach offers higher throughput and robustness without sacrificing key inflammatory features [8,44,45].

## 4. Materials and Methods

### 4.1. Materials

Cell culture reagents, including DMEM/F12 medium supplemented with stable glutamine, were purchased from VWR International (Radnor, PA, USA). Fetal bovine serum (FBS) was obtained from BioSera (Nuaille, France). Trypsin-EDTA, retinoic acid, Triton X-100, DMSO and various assay reagents were sourced from Merck (Darmstadt, Germany). Fluorescent indicators and staining dyes, including JC-1, hydroethidine (HE), 2′,7′-dichlorofluorescein diacetate (DCFDA), Mitotracker Green, Hoechst 33342, thioflavin-S, paraformaldehyde, and acridine orange (AO), were procured from ThermoFisher Scientific (Waltham, MA, USA). The Phospho-GSK-3 alpha/beta (S21/S9) DuoSet IC ELISA kit was purchased from Bio-Techne (Minneapolis, MN, USA). Insulin was supplied by the Pharmacy Department of Semmelweis University (Budapest, Hungary).

All compounds tested were dissolved in dimethyl sulfoxide (DMSO), and the final DMSO concentration in cell culture experiments was maintained below 0.5%.

### 4.2. Cell Cultures and Treatments

Human SH-SY5Y neuroblastoma cells (ECACC, Salisbury, UK) were seeded into 96-well or 24-well plates according to experimental needs and maintained in DMEM/F12 medium supplemented with 10% fetal bovine serum (FBS), 80 μg/mL gentamicin, and 10 μg/mL ciprofloxacin at 37 °C in a humidified incubator with 5% CO_2_. Cholinergic differentiation was induced by supplementing the culture with 10 µM retinoic acid (RA) on day 0, and differentiation continued for five days with medium replacement on day 3. During the differentiation period, the serum concentration was reduced to 5% to promote neuronal characteristics. Experiments were carried out using cells between passages 24 and 28.

The HL-60 cell line (ECACC, UK) was cultured in DMEM/F12 medium supplemented with 10% FBS, 80 μg/mL gentamicin and 10 μg/mL ciprofloxacin under standard culture conditions. Differentiation was carried out according to two established protocols as follows:

(1) Neutrophil-like differentiation using 1.25% DMSO [46]
(2) Monocyte-like differentiation using 100 nM RA. In both cases, differentiation was initiated on day 0 and continued for five days, with medium replacement and differentiation agent supplementation on day 3. Undifferentiated HL60 cells were also used in the experiments.

BV2 and RAW264.7 cells were cultured in DMEM/F12 medium supplemented with 10% FBS, 80 μg/mL gentamicin and 10 μg/mL ciprofloxacin under standard conditions. These cells were used without any differentiation.

On day 5 of differentiation (or after reaching 70–80% confluency in the case of undifferentiated cells), a subset of BV2, RAW264.7, and HL-60 cells (both differentiated and undifferentiated) were treated with 100 ng/mL lipopolysaccharide (LPS) for 24 h. The medium was collected by centrifugation at 500× *g* for 5 min to remove cells and debris, then filtered through a 0.22 µm syringe filter, and then diluted with fresh DMEM/F12 medium (5% FBS, 10 µM RA) at a 1:1 ratio. This conditioned medium was subsequently used to treat the differentiated SH-SY5Y cells (shown in Table 1).

### 4.3. Lactate Dehydrogenase (LDH) Release Cytotoxicity Assay

Cell viability of SH-SY5Y cells was evaluated by measuring lactate dehydrogenase (LDH) release using CytoTox-ONE Homogeneous Membrane Integrity Assay (Promega, Madison, WI, USA) as per the manufacturer’s guidelines. After treatment, supernatants were collected for LDH quantification via fluorescence at excitation and emission wavelengths of 530 and 590 nm, respectively. Data normalization was performed relative to total LDH content after complete cell lysis.

### 4.4. Assessment of ROS Levels and Mitochondrial Membrane Potential

Intracellular ROS and mitochondrial membrane potential were assessed in SH-SY5Y cells using DCFDA, hydroethidine (HE), and JC-1 staining methods. Cells were incubated with DCFDA (2 µM), HE (1 µM) and JC-1 (5 µM) for 30 min at 37 °C. Following washing with PBS, fluorescence was measured using a microplate reader at 485/530 nm for DCFDA and at 510/600 nm for HE. JC-1 signals were recorded at 485/530 nm (green) and 530/590 nm (red), and mitochondrial membrane depolarization was calculated by the green/red fluorescence ratio, with data normalization to nuclear fluorescence using Hoechst 33342.

### 4.5. Measurement of Mitochondrial Content

Mitochondrial mass was quantified in SH-SY5Y cells using Mitotracker Green dye. Cells were stained with Mitotracker Green (200 nM) for 30 min at 37 °C, washed with PBS, and fluorescence intensity was measured at 485/530 nm. Values were normalized against nuclear staining intensity using Hoechst 33342.

### 4.6. 2-NBDG Glucose Uptake Assay

After appropriate treatment SH-SY5Y cells in 96-well plates were incubated in glucose-free DMEM/F12 medium containing 100 µM 2-NBDG for 30 min at 37 °C. After incubation, cells were washed three times with ice-cold PBS, and fluorescence intensity was measured immediately using a microplate reader at excitation/emission wavelengths of 485/535 nm. The results were normalized to cell number.

### 4.7. Detection of Autophagic Vacuoles by Acridine Orange

Autophagic vacuoles in SH-SY5Y cells were detected using acridine orange staining. Cells were stained with acridine orange (1 µM) for 15 min at 37 °C in the dark. After washing, fluorescence intensity (red channel) indicative of autophagic activity was quantified using epifluorescence microscopy.

### 4.8. ELISA for Phospho-GSK-3 Alpha/Beta (S21/S9)

SH-SY5Y cells were seeded in 10 cm Petri dishes at a density of 2.5 × 10^6^ cells per dish and differentiated as described above. After differentiation, the cells were treated with the conditioned media of BV2 cells. Following 72 h of exposure, cells were treated with insulin at concentrations ranging from 10 to 1000 nM for 1 h before being harvested using a lysis buffer containing 1 mM EDTA, 0.5% Triton X-100, and 6 M urea in PBS, supplemented with phosphatase inhibitor cocktails 2 and 3.

The phosphorylated form of GSK-3 alpha/beta (S21/S9) was analyzed using a DuoSet IC ELISA kit (Bio-Techne, Minneapolis, MN, USA) according to the manufacturer’s protocol.

### 4.9. Thioflavin S Protein Aggregation Staining

Protein aggregation was assessed in SH-SY5Y cells using thioflavin S staining. Treated cells were fixed in 4% paraformaldehyde for 1 h at room temperature, washed with PBS, and incubated with 0.1% thioflavin S for 10 min in the dark. Excess dye was removed by ethanol washes, and fluorescence was observed with an epifluorescence microscope equipped with suitable filters. Microscopic images were analyzed using Fiji Image V2 software (National Institute of Health, Bethesda, MD, USA), where thresholding was applied to exclude the background signal. Subsequently, the ratio of thioflavin S (green channel) to DAPI (blue channel) fluorescence pixels was calculated, reflecting the level of aggregation normalized to the number of nuclei.

### 4.10. Statistical Evaluation

Data analysis was conducted using GraphPad Prism 8 software (La Jolla, CA, USA). Concentration response curves were constructed by nonlinear regression, and comparisons among groups were carried out using one-way ANOVA followed by Dunnett’s post hoc test. Data are expressed as the percentage of the corresponding control group. The results are shown as mean ± SD. Statistical significance was determined at *p* < 0.05.

## 5. Conclusions

In summary, we present a simplified and reproducible in vitro model for neuroinflammation-related neuronal stress with AD--like pathology. By leveraging common, easy-to-handle cell lines, such as BV2 and SH-SY5Y, and incorporating LPS-driven immune activation, this system successfully mimics aspects of the neuroinflammatory environment seen in neurodegenerative diseases, characterized by oxidative stress, mitochondrial dysfunction, and the dysregulation of autophagy, as well as impaired insulin sensitivity and the accumulation of protein aggregates. Our findings show that LPS-stimulated BV2-to-SH-SY5Y-conditioned medium transfer offers a highly robust and scalable platform suitable for large-scale compound screening. Although this in vitro system—like all simplified cell culture models—cannot fully replicate the complex intercellular network of the human brain or the dynamic progression of chronic neurodegeneration, it reliably reflects key inflammatory triggers. This model holds promise for the identification of novel therapeutics targeting neuroinflammation and its downstream effects in AD and related disorders.

## Figures and Tables

**Table 1:** Abbreviations used for immune cell-derived conditioned media.

Group	Description of Treatment
RAW264.7	Macrophage cell line-produced factors
RAW264.7 + LPS	Activated macrophage cell line-produced factors
BV2	Microglial cell line-produced factors
BV2 + LPS	Activated microglial cell line-produced factors
HL60	Undifferentiated promyeloblast cell line-produced factors
HL60 + LPS	Activated, undifferentiated promyeloblast cell line-produced factors
HL60 + DMSO	Neutrophil-like HL60 cell line-produced factors
HL60 + DMSO + LPS	Activated, neutrophil-like HL60 cell line-produced factors
HL60 + RA	Monocyte-like HL60 cell line-produced factors
HL60 + RA + LPS	Activated, monocyte-like HL60 cell line-produced factors

## Data Availability

The original contributions presented in this study are included in the article. Further inquiries can be directed to the corresponding author(s).

## Abbreviations

The following abbreviations are used in this manuscript:

2-NBDG	2-(N-(7-Nitrobenz-2-oxa-1,3-diazol-4-yl)Amino)-2-Deoxyglucose (fluorescent glucose analog)
AD	Alzheimer’s Disease
AO	Acridine Orange (fluorescent dye for acidic organelles/autophagy detection)
APP	Amyloid Precursor Protein
ATP	Adenosine Triphosphate
Aβ	Amyloid-Beta Peptide
C	Control
DAPI	4′,6-Diamidino-2-Phenylindole (nuclear stain)
DCFDA	2′,7′-Dichlorofluorescin Diacetate (indicator of hydrogen peroxide)
DMEM/F12	Dulbecco’s Modified Eagle Medium/Nutrient Mixture F-12
DMSO	Dimethyl Sulfoxide
ELISA	Enzyme-Linked Immunosorbent Assay
ER	Endoplasmic Reticulum
FBS	Fetal Bovine Serum
GLP-1	Glucagon-Like Peptide-1
GSK-3β	Glycogen Synthase Kinase-3 Beta
HE	Hydroethidine
IL-1β	Interleukin-1 Beta
IL-6	Interleukin-6
iPSC	Induced Pluripotent Stem Cell
JC-1	5,5′,6,6′-Tetrachloro-1,1′,3,3′-Tetraethylbenzimidazolylcarbocyanine Iodide
LDH	Lactate Dehydrogenase
LPS	Lipopolysaccharide
NFT	Neurofibrillary Tangle
NLRP3	NOD-, LRR-, and Pyrin Domain-Containing Protein 3
NO	Nitric Oxide
pGSK-3β	Phosphorylated Glycogen Synthase Kinase-3 Beta
PARP	Poly (ADP-Ribose) Polymerase
PBS	Phosphate-Buffered Saline
PI3K	Phosphoinositide 3-Kinase
RA	Retinoic Acid
ROS	Reactive Oxygen Species
SD	Standard Deviation
SH-SY5Y	Human Neuroblastoma Cell Line
Thioflavin S	Fluorescent dye for beta-sheet-rich protein aggregates
TNF-α	Tumor Necrosis Factor-Alpha
